# Characteristics of pivotal clinical trials of FDA-approved endovascular devices between 2000 and 2018: An interrupted time series analysis

**DOI:** 10.1017/cts.2023.10

**Published:** 2023-02-03

**Authors:** John T. Moon, Menelaos Konstantinidis, Nevon Song, Nariman Nezami, Bill S. Majdalany, Allen Herr, Gary Siskin

**Affiliations:** 1 Division of Interventional Radiology and Image-Guided Medicine, Department of Radiology and Imaging Sciences, Emory University School of Medicine, Atlanta, GA, USA; 2 Division of Biostatistics, Institute of Health Policy, Management and Evaluation, University of Toronto, Toronto, Ontario, Canada; 3 Albany Medical College, Albany, NY, USA; 4 Division of Vascular and Interventional Radiology, Department of Diagnostic Radiology and Nuclear Medicine, University of Maryland School of Medicine, Baltimore, MD, USA

**Keywords:** Regulatory approval, medical devices, policy, Food and Drug Administration

## Abstract

**Background:**

The Food and Drug Administration (FDA) reviews safety, efficacy, and the quality of medical devices through its regulatory process. The FDA Safety and Innovation Act (FDASIA) of 2012 was aimed at accelerating the regulatory process for medical devices.

**Objectives:**

The purpose of our study was to (1) quantify characteristics of pivotal clinical trials (PCTs) supporting the premarket approval of endovascular medical devices and (2) analyze trends over the last two decades in light of the FDASIA.

**Methods:**

We surveyed the study designs of endovascular devices with PCTs from the US FDA pre-market approval medical devices database. The effect of FDASIA on key design parameters (e.g., randomization, masking, and number of enrolled patients) was estimated using an interrupted time series analysis (segmented regression).

**Results:**

We identified 117 devices between 2000–2018. FDASIA was associated with a decrease in double blinding (*p* < 0.0001) and a decrease in historical comparators (*p* < 0.0001).

**Discussion:**

Our results reveal an overall trend of decreased regulatory requirements as it relates to clinical trial characteristics, but a compensatory increased rate of post-approval across device classes. Furthermore, there was an emphasis on proving equivalence or non-inferiority rather than more use of active comparators in clinical trials. Medical device stakeholders, notably clinicians, must be aware of the shifting regulatory landscape in order to play an active role in promoting patient safety.

## Introduction

The medical devices utilized in device-driven specialties, such as in Interventional Radiology (IR), Vascular Surgery, or Endovascular Neurosurgery, undergo rigorous evaluation and regulation by the US Food and Drug Administration (FDA) to ensure patient safety and clinical efficacy as part of the approval process [1].

The FDA categorizes medical devices into Class I, Class II, and Class III devices. Class I and Class II devices may apply for FDA exemption or a 510(k), which supports a device’s substantial equivalence to an existing predicate device [2]. On the other hand, Class III devices require pre-market approval (PMA) pathway, which focuses on validating the safety and efficacy of a new device. PMA is the most stringent regulatory category for medical devices and represents the umbrella category for many of the devices utilized in IR [2]. PMA is the FDA process of scientific and regulatory review for the safety and effectiveness of Class III devices, and approval is determined by “reasonable assurance” based on scientific evidence that a device is safe for its intended use(s) [2]. At baseline, preclinical data are required and validation in human clinical studies is almost always necessary. Trial data that provide key supporting evidence for the approval of medical devices are designated as pivotal clinical trials (PCTs). PCT is not an official regulatory designation but reflects trial data that were utilized for an FDA decision [3]. This study quantifies the characteristics of PCTs in approved endovascular devices commonly used in IR.

In an effort to address increasing demands for faster evaluation and approval of medical devices, the FDA has also implemented a series of policies over the years. Of these, the FDA Safety and Innovation Act (FDASIA) enacted by congress in 2012 became a focus for our study in examining pre- and post-FDASIA changes in PCT characteristics. This legislative act placed provisions in the regulatory pathway to decrease time-to-evaluation in an effort to provide faster access to novel medical technologies by decreasing threshold for granting investigational device exemptions, giving authority to collect user fees from industry to fund reviews of medical devices, allocating user-fee revenue toward reducing staff turnover, and by creating formal timelines for the FDA to respond to manufacturers’ pre-submission inquiries, all of which are seen as barriers to efficient application review [4].

This study (1) quantifies the characteristics of the PCTs supporting FDA-Approved Endovascular Devices between 2000 and 2018 via an interrupted time series (ITS) analysis and also (2) analyzes trends of PMA over the last two decades in light of FDASIA. Understanding the landscape and changes in FDA approval patterns will help all stakeholders better direct their efforts in clinical trial design to navigate a product to market optimally. The results of this retrospective study inform medical device stakeholders of both the rigor of clinical trial design and the level of evidence required for device approval prior to clinical application.

## Methods

The US FDA PMA medical device database (AccessData) was queried for medical devices between 2000 and 2018. A total of 354 medical dossiers corresponding to 117 devices were used to identify PCT characteristics focused on four key IR devices: stents, catheters, endoprosthesis, and closure devices. These devices classes are the commonly utilized device classes over the course of a procedure, which involves access (catheters), intervention (stents, endoprosthesis), and closure (vascular closure devices).

The primary characteristics quantified from the PCTs include the number of enrollees, randomization, masking type, comparator type, post-approval study requirements, and the number of arms. Subsequently, two primary scientific objectives were defined, namely, (1) characterization of approved devices with respect to the device characteristics and (2) difference in characterization before and after the FDASIA.

In characterizing the devices, the descriptive statistics of the 117 devices are presented with respect to the variables of interest stratified by device class. Additionally, the characteristics of different device categories are compared. For categorical variables (randomization, masking type, comparator type, and post-approval study requirements, number of arms), an exact fisher test of independence was utilized. For the remaining variables (number of enrollees and number of arms), a Kruskal–Wallis Chi-squared test was conducted.

In addressing the effects of the FDASIA, an ITS analysis [5–7] was used for each device characteristic (separately) through a segmented regression, modeling the effect of the FDASIA on a given device characteristic (i.e., randomization, masking, etc.). Such an analysis allowed for the estimation of the effect of FDASIA while accounting for possible cohort effects and observed confounders (in this case, the device class). For binary outcomes (i.e., randomization and post-study approval) and multi-category outcomes (i.e., Masking and Comparators), this was done through a segmented binomial logistic regression and multinomial logistic regression, respectively. For continuous outcome variables, this was done through a segmented Poisson regression. Model adequacy was tested against the Akaike information criteria [8]. In each analysis, autocorrelation was tested by visual inspection of the residuals and by the Durbin–Watson test. Moreover, stationarity was tested by visual inspection of the autocorrelation functions.

## Results

### Device Characterization

**Fig. 1.**
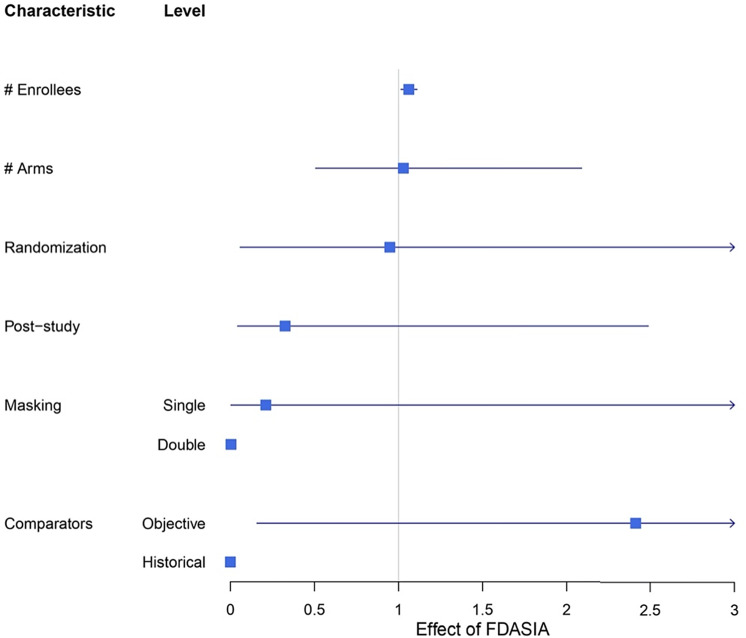
Forest plot of effect estimates from interrupted time series analyses with line of no effect (i.e., null hypothesis) at 1 both for categorical variables and count variables corresponding to logistic and poisson regression.

General characterization of 117 endovascular devices with respect to clinical trial characteristics are described in Table 1. Requirements for randomization across device classes were found to be different (*p* < 0.0001), with catheters having the lowest rate of randomization (Table 1 and Fig. 1). Statistically significant differences were also found between the proportion of devices requiring types of comparators (*p* < 0.001). Most notably, closure devices at 100% active comparators, whereas other device classes at least one-quarter of their studies conducted with objective performance criterion. Furthermore, statistically significant differences were found between the proportion of post-study approval studies (*p* < 0.0001). In particular, higher rates of post-approval studies for stents, endoprosthesis, and catheter devices were shown compared to 10% of closure devices requiring post-approval studies.

**Table 1.** Characteristics of pivotal pre-approval trials for endovascular devices between 2000 and 2018

	Stent (*n* = 77)	Endoprosthesis (*n* = 7)	Closure (*n* = 10)	Catheter (*n* = 24)	*p*-value
Enrollees (#)					
Mean (SD)	409.27 (397.43)	290.00 (322.88)	401.80 (175.97)	323.20 (218.83)	0.36
Randomization (%)					
Prospective, non-randomized	71.43	71.40	100.00	52.17	<0.0001
Prospective, randomized	28.57	28.60	0.00	47.83	
Masking (%)					
None (open label)	77.90	71.43	100.00	82.61	0.67
Single (participant)	18.20	28.57	0.00	17.39	
Double-blind	3.90	0.00	0.00	0.00	
Comparators (%)					
Active comparator	40.26	57.14	100.00	43.48	<0.001
Objective performance criterion	45.45	28.57	0.00	34.78	
Historical control	14.29	14.29	0.00	21.74	
Arms					
Mean (SD)	1.30 (0.46)	1.57 (0.54)	2 (0)	1.44 (0.51)	<0.001
Post study (%)					
No	14.29	28.57	90.00	43.48	<0.0001
Yes	85.71	71.43	10.00	56.52	

### FDASIA

The descriptive statistics for the devices pre-FDASIA and post-FDASIA provide a basis for comparison in the characterization of these endovascular devices before and after the legislative act with respect to clinical trial characteristics (Table 2). Post-FDASIA, there was a 2.89% increase (from 45.95%) in the number of studies utilizing active comparators, a 1.7% increase (from 37.84%) in studies utilizing objective performance criterion, and a 4.59% decrease (from 16.22%) in studies utilizing historical controls. Post-FDASIA, there was a decrease in the number of enrollees (419.31–324.79), an increase in open-label studies (75.68%–88.37%), and a decrease of double-blinded studies (4.05%–0.00%). There was also a higher proportion of devices requiring post-approval studies after FDASIA, from 66.22% to 81.40%.

**Table 2.** Characteristics of pivotal pre-approval trials for endovascular devices pre- and post-FDASIA

	Pre-FDASIA (*n* = 74)	Post-FDASIA (*n* = 43)
Enrollees (#)		
Mean (SD)	419.31 (403.62)	324.79 (201.32)
Randomization (%)		
Prospective, non-randomized	63.51	58.14
Prospective, randomized	36.49	41.86
Masking (%)		
None (open label)	75.68	88.37
Single (participant)	20.27	11.63
Double-blind	4.05	0.00
Comparators (%)		
Active comparator	45.95	48.84
Objective performance criterion	37.84	39.54
Historical control	16.22	11.63
Arms (#)		
Mean (SD)	1.37 (0.49)	1.47 (0.51)
Post study (%)		
No	33.78	18.61
Yes	66.22	81.40

FDASIA, Food and Drug Administration Safety and Innovation Act.

The FDASIA was found to reduce masking requirements from 4% to 0%, such that, relative to no blinding, FDASIA was associated with a decrease in the odds of a device being double-blinded (*p* < 0.0001). FDASIA was also associated with a decrease in the proportion of a device being single-blinded; however, it was not statistically significant (*p* = 0.47) (Fig. 1).

Additionally, the effect of FDASIA on comparators was heterogeneous. Relative to “Active” comparators, FDASIA was associated with a decrease in the odds of a device having a historical comparator (*p* < 0.0001) and, while not statistically significant, was associated with an increase in the odds of a device having an objective comparator (*p* < 0.53) (Fig. 1). Furthermore, FDASIA was associated with a decrease in the number of enrollees (*p* < 0.01) (Fig. 1).

FDASIA had no statistically detectable difference in the odds of randomization (*p* = 0.97) or post-study approval requirements (*p* = 0.28) (Fig. 1). Lastly, FDASIA had no statistically significant effect on the number of arms (*p* = 0.84).

## Discussion

In characterizing endovascular devices with respect to clinical trial characteristics, our findings showed that closure device trials were 100% prospective and non-randomized, while stents and endoprosthesis had an approximate 70%–30% ratio of prospective, non-randomized to prospective, randomized trials. This predominance of prospective, non-randomized clinical trials may not be surprising if a new device is aimed at proving equivalence or non-inferiority. These devices are aimed at providing similar or improved effectiveness to existing standards but with fewer side effects, greater convenience, or lower cost [9]. Additionally, fewer resources and ensuing costs may be required for noninferiority studies as compared to superiority studies. To qualify, the trend towards non-inferiority studies, and their less rigorous design, is not necessarily justified due to their practical benefits. In fact, this likely makes the evidence supporting devices less reliable by physicians and patients. Finally, higher rates of post-approval studies for stents, endoprosthesis, and catheter devices, as compared to closure devices, are likely because these devices remain within the body for an extended amount of time and may require increased post-market surveillance.

Our study also examined clinical trial characteristics both before and after the FDASIA implementation. The FDASIA was intended to re-regulate aspects of the de novo 510(k) pathway, device classification, appeals, modifications, reclassification, as well as the IDE process. These changes were aimed to encourage innovation, decrease time to evaluation, and decrease time to approval as a result of less stringent clinical trial requirements [10]. Our results showed that clinical trials decreased double-blinded studies from 4% to 0%; however, more strikingly greater than 95% of studies remain unblinded following FDASIA. Unblinded studies are likely a function of cost as double-blinded studies typically take several months longer and may not be a practical approach for the intrinsic properties of endovascular devices. In fact, the decrease in number of enrollees has paralleled a decrease in number of experimental arms and blinding used in clinical trials. Again, this trend is observed and not justified in the less rigorous data provided to patients and clinicians. Thus, balancing the rigor of a clinical trial against anticipated time to completion and subsequent costs is important in assuring clinically relevant data while accelerating potential device availability.

Following FDASIA, there was less use of historical comparators compared to active comparators. Historical controls may address the problem of sample size but provide an incomplete comparison of a device. Temporal changes in clinical practice, more developed procedural and patient management experience, and changes in the baseline characteristics of a patient cohort can all raise concerns over the validity of historical comparators [10]. Active comparators, on the other hand, increase the overlap of measured characteristics between patient groups, and study designs involving active comparators also reduce differences in unmeasured pretreatment characteristics and potential confounding, which cannot be easily accounted for [11]. Our characterization of these devices over time supports a trend towards non-inferiority. Policy implementation towards accelerated development must be coupled with the value derived from reported data and their impact on physician and patient choices, and ultimately long-term outcomes of utilized devices.

Further monitoring and a larger sample size over time may show that FDASIA, having decreased time to approval for endovascular devices, may not necessarily have increased requirements to monitor these devices once they are in the market. We do not include the data regarding completed post-approval studies of the devices in this study as it is not yet fully available. Investigating the shift of regulatory and cost burden from pre- to post-approval will be crucial to determining the effects of high costs and limited value of post-approval studies on driving device approval. Enforcing post-market surveillance and continued FDA oversight may be important to consider for device safety, especially given known challenges in the drug industry [12]. Post-market studies have, in general, faced issues with recruiting enough patients, loss to follow-up, consistent reporting, and timely completion [13]. The FDA has, since FDASIA, proposed new strategies to improve post-market safety assessments of medical devices, but with unclear results or limited demonstrated value [14]. In addition, it is estimated that more than $1.22 billion is planned to be spent on FDA-mandated post-approval studies for medical devices approved between March 2005 and June 2013, with 50% of those devices requiring post-approval studies being cardiovascular devices. It is vital for post-approval to be designed to lower costs and more effectively monitor the safety and effectiveness of these devices once on the market [14].

Despite the added value of the present results, our study is not without limitations. In particular, despite the relatively large sample size arrived at from the survey of PCTs, the sample size of endoprosthesis and closure devices was small (7 and 10, respectively); stratification or adjustment was therefore not reliably implemented – a possible confounding variable. Moreover, because of the retrospective nature of the dataset, additional confounders were not consistently reported (and consequently not accounted for), thus potentially biasing the present results. Additionally, for the purpose of the present analysis, it was assumed that FDASIA would have had an approximately immediate effect on changes in study designs; however, this may not necessarily be the case and should be explored further in future work.

## Supplementary material

For supplementary material accompanying this paper visit https://doi.org/10.1017/cts.2023.10. click here to view supplementary material
